# The genome sequence of the Shaded Fan-foot, *Herminia tarsicrinalis* (Knoch, 1782) (Lepidoptera: Erebidae)

**DOI:** 10.12688/wellcomeopenres.27808.1

**Published:** 2026-09-14

**Authors:** oreina, Papillons de France, Antonio S. Ortiz, Charlotte J. Wright, Joana I. Meier, Mark L. Blaxter, Wellcome Sanger Institute Tree of Life Management, Samples and Laboratory team, Wellcome Sanger Institute Scientific Operations: Sequencing Operations, Wellcome Sanger Institute Tree of Life Core Informatics team, Tree of Life Core Informatics collective, Project Psyche Community

**Affiliations:** 1 Oreina, Thoury-Férottes (Seine-et-Marne), France; 2 Department of Zoology and Physical Anthropology, University of Murcia, Murcia, Spain; 3 Tree of Life, Wellcome Sanger Institute, Hinxton, England, UK

**Keywords:** Herminia tarsicrinalis, Shaded Fan-foot, genome sequence, chromosomal, Lepidoptera

## Abstract

We present a genome assembly from a female specimen of *Herminia tarsicrinalis* (Shaded Fan-foot; Arthropoda; Insecta; Lepidoptera; Erebidae). The assembly contains two haplotypes with total lengths of 801.83 megabases and 720.01 megabases. Most of the haplotype 1 assembly (99.02%) was scaffolded into 32 chromosomal pseudomolecules, including the W and Z sex chromosomes. Haplotype 2 was assembled to scaffold level. The mitochondrial genome has also been assembled, with a length of 16.98 kilobases. Gene annotation of this assembly by Ensembl identified 12,268 protein-coding genes. This work is part of Project Psyche, a collaborative programme generating genomes for European butterflies and moths.

## Species taxonomy

Eukaryota; Opisthokonta; Metazoa; Eumetazoa; Bilateria; Protostomia; Ecdysozoa; Panarthropoda; Arthropoda; Mandibulata; Pancrustacea; Altocrustacea; Allotriocarida; Hexapoda; Insecta; Dicondylia; Pterygota; Neoptera; Eumetabola; Endopterygota; Aparaglossata; Panorpida; Amphiesmenoptera; Lepidoptera; Glossata; Neolepidoptera; Heteroneura; Ditrysia; Obtectomera; Noctuoidea; Erebidae; Herminiinae; *Herminia*; *Herminia tarsicrinalis* (Knoch, 1782) (NCBI:txid938191)

## Background

*Herminia tarsicrinalis* (Knoch, 1782), commonly known as the Shaded Fan-foot, is a medium-sized noctuid moth with a wingspan of between 19 and 35 mm. The species is characterised by a uniformly pale brown head, thorax, abdomen and ground colour, sometimes with a greyish tinge. Labial palpi are long, slender and curved. The forewing is quite broad, with antemedial and postmedial lines and the reniform spot well marked; the antemedial line is slightly curved, the postmedial line sinuous, and the anteterminal line almost straight and not reaching the tip of the costa. The hindwing is lighter coloured, with lines barely visible.

The species flies in one generation from June to August and, at low latitudes, in two generations, flying until October. Larvae are polyphagous, feeding on withered leaves of a number of woody plants such as *Rubus fruticosus* and *Clematis* spp. *Herminia tarsicrinalis* inhabits humid and subhumid woodland areas. A trans-Palaearctic species, it is distributed from Europe to Korea, Japan and China ( Fibiger *et al.*, 2010).

Genetically, the European species is characterised by the Barcode Index Number (BIN) BOLD:AAC1537, calculated with 54 sequences and an average intraspecific pairwise divergence in COI-5P of 0.17% (maximum divergence 0.65%) ( Ratnasingham & Hebert, 2007).

The genome sequence of this species will be fundamental for understanding the systematic relationships with species of the subfamily Herminiinae in the genera *Paracolax* Hübner, 1825, *Zanclognatha* Lederer, 1857, *Pechipogo* Hübner, 1825 and *Polypogon* Schrank, 1802. At the moment, only the genome of *Polypogon plumigeralis* (Hübner, 1825) has been sequenced ( Lees *et al.*, 2025).

We present a chromosome-level, haplotype-resolved genome sequence of the Shaded Fan-foot, *Herminia tarsicrinalis*, sequenced as part of Project Psyche ( Wright *et al.*, 2025). The sequence data were derived from a specimen (Figure 1) collected from Saint-Étienne-de-Baïgorry, Pyrénées-Atlantiques, Pays Basque, France.

**Figure 1.**
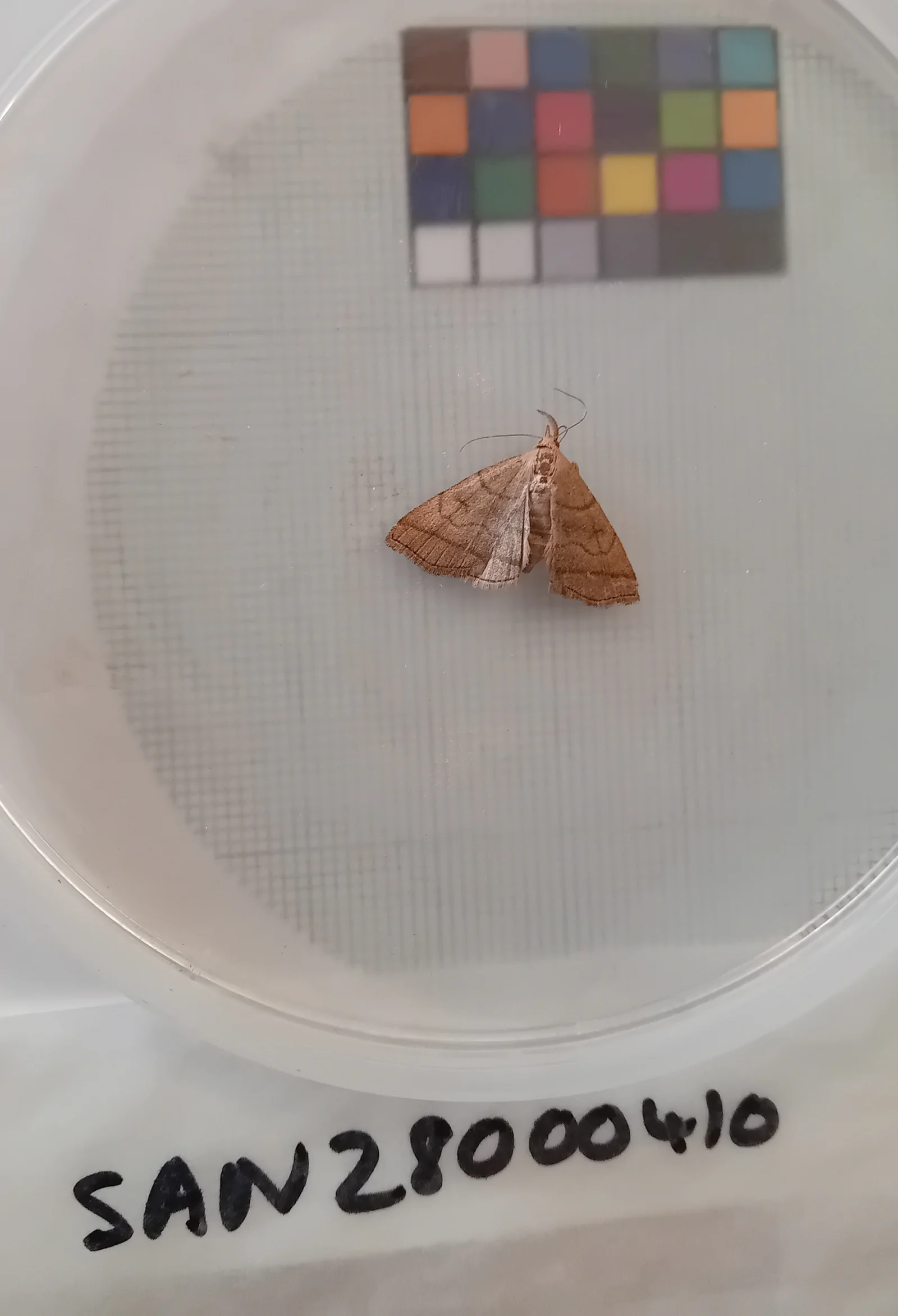
Voucher photograph of the *Herminia tarsicrinalis* specimen (ilHerTars1) used for genome sequencing.

## Methods

### Sample acquisition

The specimen used for genome sequencing was an adult female *Herminia tarsicrinalis* (specimen ID SAN28000410, ToLID ilHerTars1; Figure 1), collected from Saint-Étienne-de-Baïgorry, Pyrénées-Atlantiques, Pays Basque, France (latitude 43.1814, longitude −1.3739) on 2024-05-18. The specimen was collected and identified by members of *oreina*, Papillons de France.

### Nucleic acid extraction

Protocols for high molecular weight (HMW) DNA extraction developed at the Wellcome Sanger Institute (WSI) Tree of Life Core Laboratory are available on protocols.io ( Howard *et al.*, 2025). For HMW DNA extraction, tissue from the thorax of ilHerTars1 was homogenised by powermashing using a PowerMasher II tissue disruptor. HMW DNA was extracted in the WSI Scientific Operations core using the Automated MagAttract v2 protocol. DNA was sheared into an average fragment size of 12–20 kb following the Megaruptor®3 for LI PacBio protocol. Sheared DNA was purified by automated SPRI (solid-phase reversible immobilisation). The concentration of the sheared and purified DNA was assessed using a Nanodrop spectrophotometer and Qubit Fluorometer using the Qubit dsDNA High Sensitivity Assay kit. Fragment size distribution was evaluated by running the sample on the Femto Pulse system. For this sample, the final post-shearing DNA had a Qubit concentration of 29.85 ng/μL and a yield of 1,402.95 ng, with a fragment size of 17.5 kb. The 260/280 spectrophotometric ratio was 1.77, and the 260/230 ratio was 2.2. The Genomic Quality Number (GQN) was 7.6.

### PacBio HiFi library preparation and sequencing

Library preparation and sequencing took place in the WSI Scientific Operations core. Library PSYCHE15123099 was prepared with the SMRTbell Prep Kit 3.0 (Pacific Biosciences) according to the manufacturer’s instructions. The kit includes reagents for end repair/A-tailing, adapter ligation, post-ligation SMRTbell bead clean-up, and nuclease treatment. Size selection and clean-up were performed using diluted AMPure PB beads (Pacific Biosciences). DNA concentration was quantified with a Qubit Fluorometer v4.0 (Thermo Fisher Scientific) and the Qubit 1X dsDNA HS assay kit. Final library fragment size was assessed with the Agilent Femto Pulse Automated Pulsed Field CE Instrument (Agilent Technologies) using the gDNA 55 kb BAC analysis kit.

The library was sequenced on a Revio instrument (Pacific Biosciences, California, USA). Prepared libraries with a molarity greater than 2 nM were normalised to 2 nM, and 15 μL was used to make complexes. For libraries with a molarity below 2 nM, the available 10 μL library volume was used without normalisation. Primers were annealed and polymerases were bound to generate circularised complexes following the manufacturer’s instructions. Complexes were purified using 1.2× SMRTbell beads, diluted to a loading concentration of 200–300 pM, and spiked with a Revio sequencing internal control. Sequencing used Revio 25M SMRT cells. SMRT Link software was used to configure and monitor the run and to perform primary and secondary data analysis.

### Hi-C

***Sample preparation and crosslinking***

Hi-C data were generated from the ilHerTars1 head sample using the Arima-HiC v2 kit (Arima Genomics). Following the manufacturer’s instructions, tissue was fixed and DNA crosslinked using TC buffer to a final formaldehyde concentration of 2%. The tissue was homogenised using the Diagnocine Power Masher-II. Crosslinked DNA was digested with a restriction enzyme master mix, biotinylated, and ligated. Clean-up was performed with SPRISelect beads before library preparation. DNA concentration was measured with the Qubit Fluorometer (Thermo Fisher Scientific) and Qubit HS Assay Kit. The biotinylation percentage was estimated using the Arima-HiC v2 QC beads.

***Hi-C library preparation and sequencing***

Biotinylated DNA constructs were fragmented using a Covaris E220 sonicator and size-selected to 400–600 bp using SPRISelect beads. DNA was enriched with Arima-HiC v2 kit Enrichment beads. End repair, A-tailing, and adapter ligation were carried out with the NEBNext Ultra II DNA Library Prep Kit (New England Biolabs), following a modified protocol where library preparation occurs while DNA remains bound to the Enrichment beads. Libraries were amplified with KAPA HiFi HotStart mix and a custom Unique Dual Index (UDI) barcode set (Integrated DNA Technologies). Depending on sample concentration and biotinylation percentage determined at the crosslinking stage, libraries were amplified with 10–16 PCR cycles. Post-PCR clean-up was performed with SPRISelect beads. Libraries were quantified using the AccuClear Ultra High Sensitivity dsDNA Standards Assay Kit (Biotium) and a FLUOstar Omega plate reader (BMG Labtech).

Prior to sequencing, libraries were normalised to 10 ng/μL. Normalised libraries were quantified again to create equimolar and/or weighted 2.8 nM pools. Pool concentrations were checked using the Agilent 4200 TapeStation (Agilent) with High Sensitivity D500 reagents before sequencing. Libraries were sequenced on the Illumina NovaSeq X, generating paired-end 150-bp reads.

### Genome assembly

Prior to assembly of the PacBio HiFi reads, a database of *k*-mer counts ( *k* = 31) was generated from the filtered reads using FastK. The *k*-mer frequency distribution was analysed with GenomeScope (version 2.1.0) ( Ranallo-Benavidez *et al.*, 2020), providing fitted point estimates of genome size, heterozygosity and repeat content.

The HiFi reads were assembled using Hifiasm in Hi-C phasing mode ( Cheng *et al.*, 2021, 2022), producing two haplotypes. Hi-C reads ( Rao *et al.* , 2014) were mapped to contigs using bwa-mem2 ( Vasimuddin *et al.*, 2019). Contigs were further scaffolded with Hi-C data in YaHS ( Zhou *et al.*, 2023), using the --break option for handling potential misassemblies. The scaffolded assemblies were evaluated using Gfastats ( Formenti *et al.* , 2022), BUSCO ( Manni *et al.*, 2021) and MerquryFK ( Rhie *et al.*, 2020).

The mitochondrial genome was assembled using MitoHiFi ( Uliano-Silva *et al.* , 2023), which runs MitoFinder ( Allio *et al.*, 2020) and uses these annotations to select the final mitochondrial contig and to ensure the general quality of the sequence, using the mitochondrial genome of *Hydrillodes lentalis* (NC_041106.1) as the reference.

### Assembly curation

The assembly was decontaminated using the Assembly Screen for Cobionts and Contaminants ( ASCC) pipeline. The flat files and maps for curation were generated with TreeVal. Manual curation was conducted primarily in PretextView and HiGlass ( Kerpedjiev *et al.* , 2018). Scaffolds were visually inspected and corrected as described by Howe *et al*. (2021). The two haplotype assemblies were combined for curation. Manual corrections included eight breaks and 151 joins. This reduced the scaffold count by 27.4%, increased the scaffold N50 by 0.8%, and increased the total assembly length by 1.6%. The curation process is described at sanger-tol/curation-resources. A Hi-C contact map of the haplotype 1 assembly was generated with PretextSnapshot.

### Assembly quality assessment

The MerquryFK tool ( Rhie *et al.*, 2020), run in a Singularity container ( Kurtzer *et al.*, 2017), was used to evaluate *k*-mer completeness and assembly quality for both haplotypes using the *k*-mer databases ( *k* = 31) computed prior to genome assembly. The analysis outputs included assembly QV scores and completeness statistics.

The haplotype 1 assembly was assessed using BlobToolKit ( Challis *et al.*, 2020). PacBio reads were mapped to the haplotype 1 assembly to derive sequence coverage, and BUSCO ( Manni *et al.*, 2021) was used to assess gene-set completeness. DIAMOND BLASTp searches of extracted BUSCO proteins and DIAMOND BLASTx searches of assembly sequences against UniProt Reference Proteomes ( Buchfink *et al.*, 2021), followed by BLASTn searches of sequences without DIAMOND BLASTx hits against the NCBI nt database ( Altschul *et al.*, 1990), supported taxonomic assignment. Coverage, sequence composition, taxonomic assignment and BUSCO results were integrated into a BlobDir for interactive inspection and visualisation. The snail and blob plots were rendered locally from this hosted BlobDir using BlobTk v0.8.3. The scaffold-length radial scale function for the snail plot was set to linear ( Challis & Blaxter, 2026). The blob plot used circular markers with area proportional to scaffold length ( Challis *et al.*, 2020).

We used *busco-alg-painter* v0.1.0, a Python reimplementation and extension of *lep_busco_painter*, to paint Merian elements along chromosomes. Merian elements represent the 32 ancestral linkage groups reconstructed for Lepidoptera ( Wright *et al.*, 2024). The BUSCO-to-Merian reference assignments were derived from *lep_busco_painter*. Complete single-copy and duplicated BUSCO genes from the lepidoptera_odb10 results were assigned to Merian elements. Chromosome identifiers and lengths were obtained from NCBI Datasets, and BUSCO positions were plotted on chromosomes drawn to scale.

## Genome sequence report

### Sequence data

PacBio sequencing of the *H. tarsicrinalis* specimen generated 90.83 Gb (gigabases) from 8.41 million reads. We assembled the genome from these reads. GenomeScope analysis using a *p* = 2 model gave a *k*-mer-based haploid genome size estimate of 750.10 Mb, with heterozygosity of 2.33% and repeat content of 38.77%; the full-model fit was 94.81% (Figure 2). The GenomeScope estimates guided expectations for the assembly. The sequencing data provided approximately 117× coverage of the haplotype 1 assembly. Hi-C sequencing produced 130.93 Gb from 433.54 million reads, which were used to scaffold the haplotype 1 assembly. Table 1 summarises the specimen and sequencing details.

**Figure 2.**
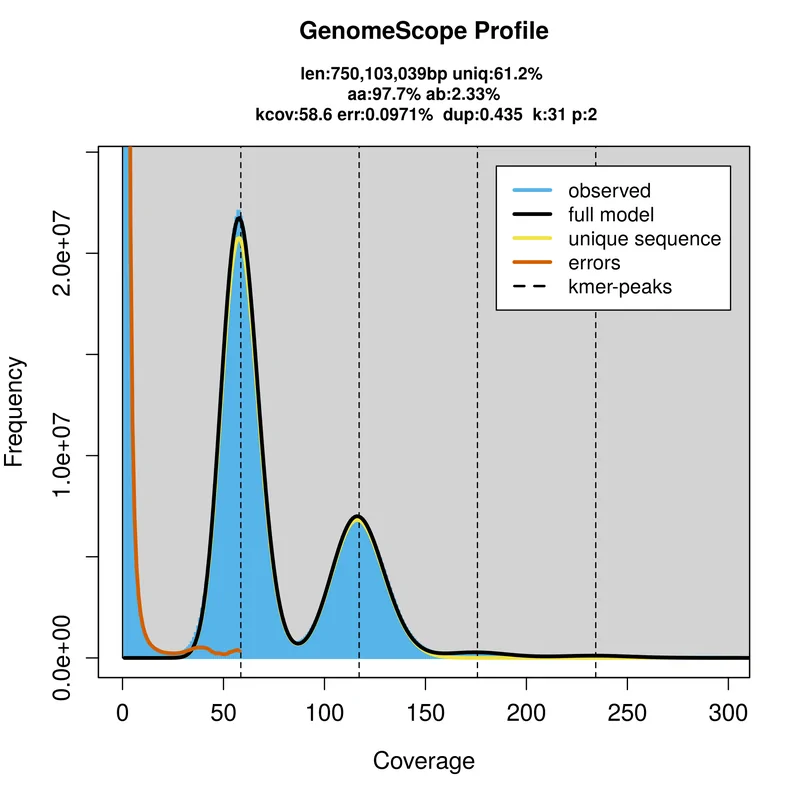
GenomeScope model fitted to the *k*-mer frequency distribution. The plot shows the observed and modelled *k*-mer spectra used to estimate genome size, heterozygosity and repeat content from unassembled sequencing reads.

**Table 1.** Specimen and sequencing data for *H. tarsicrinalis* (BioProject PRJEB82330)

**Platform**	**PacBio HiFi**	**Hi-C**
**ToLID**	ilHerTars1	ilHerTars1
**Specimen ID**	SAN28000410	SAN28000410
**BioSample (source individual)**	SAMEA115948962	SAMEA115948962
**BioSample (tissue)**	SAMEA115949475	SAMEA115949473
**Tissue**	thorax	head
**Instrument**	Revio (1 run)	Illumina NovaSeq X
**Library ID & run accession(s)**	PSYCHE15123099: ERR13946513	ERR13947542
**Read count total**	8.41 million reads	433.54 million read pairs
**Base count total**	90.83 Gb	130.93 Gb

### Assembly statistics

The genome was assembled into two haplotypes using Hi-C phasing. The haplotype 1 assembly was curated to chromosome level, while haplotype 2 was assembled to scaffold level. The haplotype 1 assembly, which is proposed as the reference assembly for this species, has a total length of 801.83 Mb in 206 scaffolds (excluding organelle sequences), with 241 gaps, and a scaffold N50 of 25.74 Mb (Table 2). The haplotype 1 assembly has a scaffold auN of 25.8 Mb.

**Table 2.** Genome assembly statistics for *H. tarsicrinalis*

**Genome assembly**	**Haplotype 1**	**Haplotype 2**
**Assembly name**	ilHerTars1.hap1.1	ilHerTars1.hap2.1
**Assembly accession**	GCA_964656175.1	GCA_964656245.1
**Assembly level**	chromosome	scaffold
**Span (Mb)**	801.83	720.01
**Number of chromosomes**	32	-
**Number of contigs**	447	234
**Contig N50 (Mb)**	9.59	11.21
**Number of scaffolds (excluding organelle sequences)**	206	156
**Scaffold N50 (Mb)**	25.74	24.8
**Scaffold auN (Mb)**	25.8	24.1
**Longest scaffold length (Mb)**	41.53	31.38
**Sex chromosomes**	W and Z	-
**Organelles**	Mitochondrial genome: 16.98 kb	-

Most of the haplotype 1 assembly sequence (99.02%) was assigned to 32 chromosome-level scaffolds, representing 30 autosomes and the W and Z sex chromosomes. These chromosome-level scaffolds, confirmed by Hi-C data, are named according to size (Figure 3; Table 3). Chromosome painting of the haplotype 1 assembly with Merian elements illustrates the distribution of orthologues along chromosomes and highlights patterns of chromosomal evolution relative to Lepidopteran ancestral linkage groups (Figure 4). The Z and W chromosomes were assigned based on copy number in the diploid assembly and the Hi-C signal.

The mitochondrial genome was also assembled (length 16.98 kb, OZ209669.1). This sequence is included as a contig in the multifasta file of the haplotype 1 genome submission and as a standalone record.

**Figure 3.**
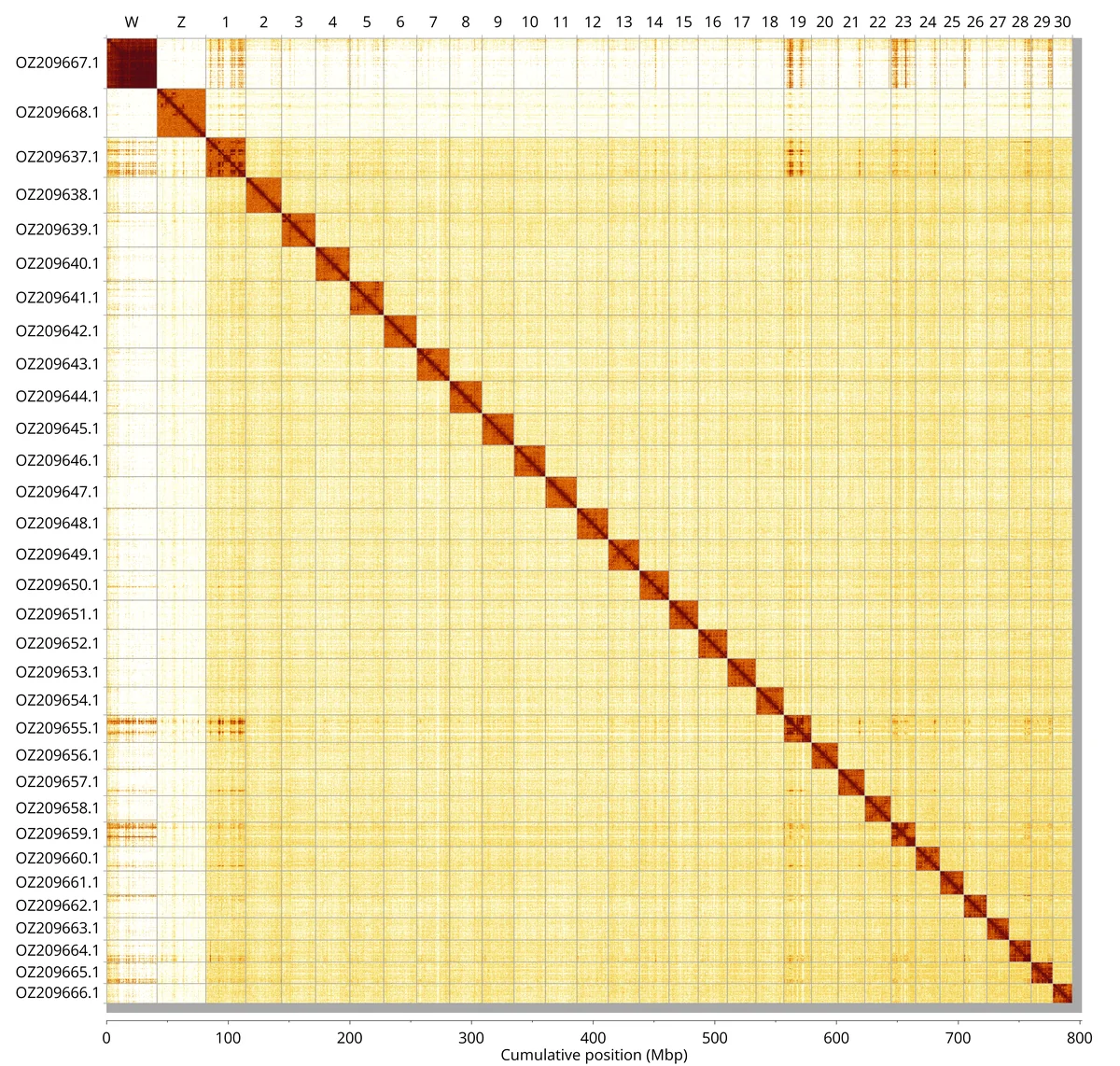
Hi-C contact map of the *H. tarsicrinalis* haplotype 1 assembly. Assembled chromosomes are shown in order of size and labelled along the axes. The plot was generated using PretextSnapshot.

**Table 3.** Chromosomal pseudomolecules in the haplotype 1 genome assembly of *H. tarsicrinalis* ilHerTars1

**INSDC accession**	**Molecule**	**Length (Mb)**	**GC%**	**Assigned Merian elements**
OZ209637.1	1	32.86	37	M23
OZ209638.1	2	29.30	37	M5
OZ209639.1	3	28.09	37	M17; M20
OZ209640.1	4	28.03	36.50	M9
OZ209641.1	5	27.91	37	M6
OZ209642.1	6	27.16	37	M2
OZ209643.1	7	27.05	37	M18
OZ209644.1	8	26.48	36.50	M8
OZ209645.1	9	26.26	37	M7
OZ209646.1	10	26	37	M3
OZ209647.1	11	25.80	37	M1
OZ209648.1	12	25.74	36.50	M12
OZ209649.1	13	25.55	37	M22
OZ209650.1	14	24.55	37	M15
OZ209651.1	15	23.93	36.50	M16
OZ209652.1	16	23.79	37	M21
OZ209653.1	17	23.59	37	M10
OZ209654.1	18	22.89	37	M4
OZ209655.1	19	22.75	38.50	M30
OZ209656.1	20	21.92	37.50	M11
OZ209657.1	21	21.76	37	M13
OZ209658.1	22	21.61	37.50	M14
OZ209659.1	23	20.30	38.50	M31
OZ209660.1	24	20.03	37.50	M19
OZ209661.1	25	19.57	37.50	M24
OZ209662.1	26	18.81	37.50	M28
OZ209663.1	27	18.41	37.50	M26
OZ209664.1	28	18.14	38	M25
OZ209665.1	29	17.65	38.50	M29
OZ209666.1	30	16.25	37.50	M27
OZ209667.1	W	41.53	38.50	-
OZ209668.1	Z	40.27	36.50	MZ

**Figure 4.**
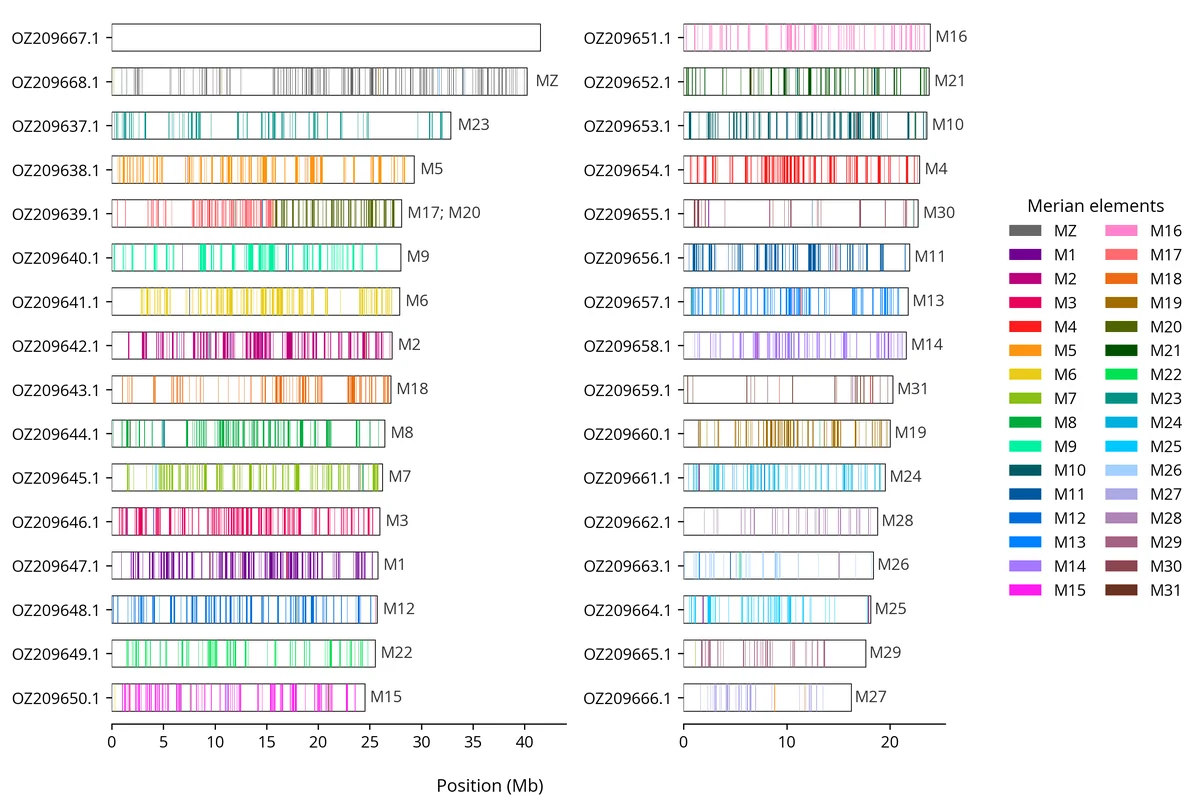
Merian elements painted across chromosomes in the ilHerTars1.hap1.1 haplotype 1 assembly of *H. tarsicrinalis*. Chromosomes are drawn to scale, with the positions of orthologues shown as coloured bars. Each orthologue is coloured by the Merian element that it belongs to. All orthologues which could be assigned to Merian elements are shown.

### Assembly quality metrics

For the haplotype 1 assembly, the estimated QV is 67.7, and for the haplotype 2 assembly, 68.4. When the two haplotypes are combined, the assembly achieves an estimated QV of 68.0. The *k*-mer completeness is 68.43% for the haplotype 1 assembly, 64.65% for the haplotype 2 assembly, and 99.65% for the combined haplotypes (Figure 5).

**Figure 5.**
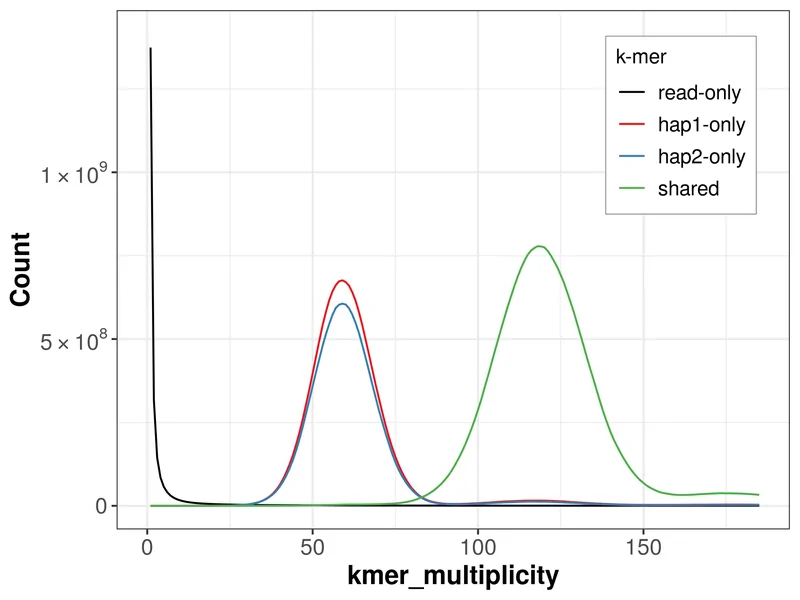
Evaluation of *k*-mer completeness using MerquryFK. This plot illustrates the recovery of *k*‐mers from the original read data in the haplotype 1 assembly, the haplotype 2 assembly and the combined haplotypes. The horizontal axis represents *k*‐mer multiplicity, and the vertical axis shows the number of *k*‐mers. The black curve represents *k*‐mers that appear in the reads but are not assembled. The green curve corresponds to *k*‐mers shared by both haplotypes, and the red and blue curves show *k*‐mers found only in one of the haplotypes.

BUSCO analysis using the lepidoptera_odb10 reference set ( *n* = 5,286) identified 98.8% of the expected gene set (single = 97.6%, duplicated = 1.2%) in the haplotype 1 assembly. For the haplotype 2 assembly, BUSCO analysis identified 94.4% of the expected gene set (single = 93.2%, duplicated = 1.3%).

The snail plot in Figure 6 provides a summary of assembly contiguity, cumulative scaffold count, sequence composition and BUSCO completeness for the haplotype 1 assembly ( Challis & Blaxter, 2026). The relative sizes of the longest scaffold, scaffold N50 and N90, together with the dark-grey scaffold-length distribution, show how much of the assembly is contained in long scaffolds. The outer composition track shows variation in GC, AT and N content, while the central track shows the cumulative scaffold count and the BUSCO inset summarises gene-set completeness.

The blob plot in Figure 7 displays scaffolds from the haplotype 1 assembly by GC proportion and base coverage. Marker area is proportional to scaffold length, and colour indicates taxonomic assignment ( Challis *et al.*, 2020).

**Figure 6.**
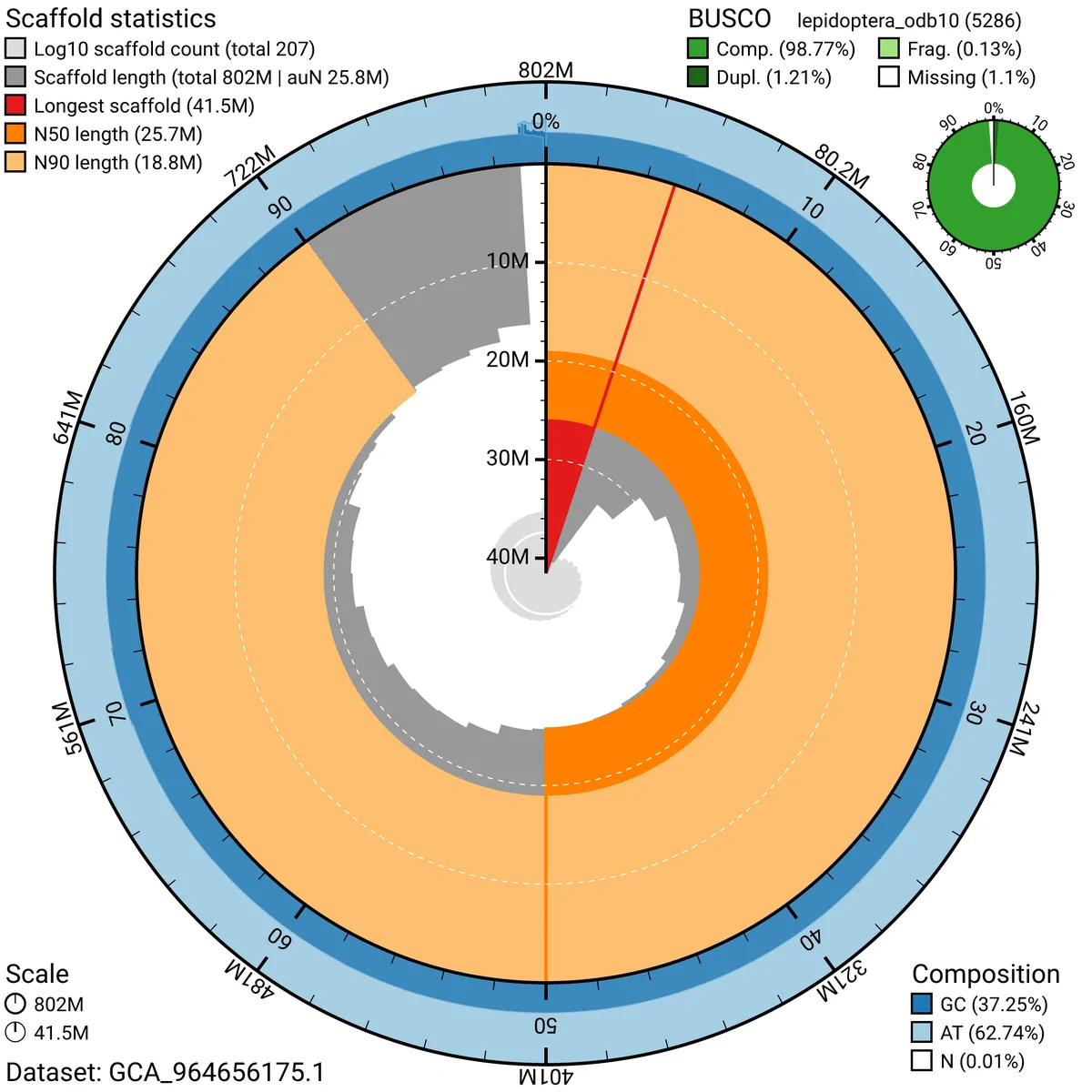
Assembly metrics for ilHerTars1.hap1.1, the haplotype 1 assembly. The BlobToolKit snail plot provides an overview of assembly metrics and BUSCO gene completeness. The circumference represents the length of the haplotype 1 assembly, and the main plot is divided into 1,000 bins around the circumference. The outermost blue tracks display the distribution of GC, AT, and N percentages across the bins. Scaffolds are arranged clockwise from longest to shortest and are depicted in dark grey. Scaffold length is shown on a linear radial scale, scaled to the longest scaffold. Red shading indicates the longest scaffold, and the deeper orange and pale orange shading represent the N50 and N90 lengths. The summary statistics include scaffold auN, the area under the scaffold Nx curve. The light grey spiral at the centre shows the cumulative scaffold count on a logarithmic scale. A summary of complete, fragmented, duplicated, and missing BUSCO genes in the lepidoptera_odb10 set is presented at the top right. The assembly can be explored interactively in the BlobToolKit viewer.

**Figure 7.**
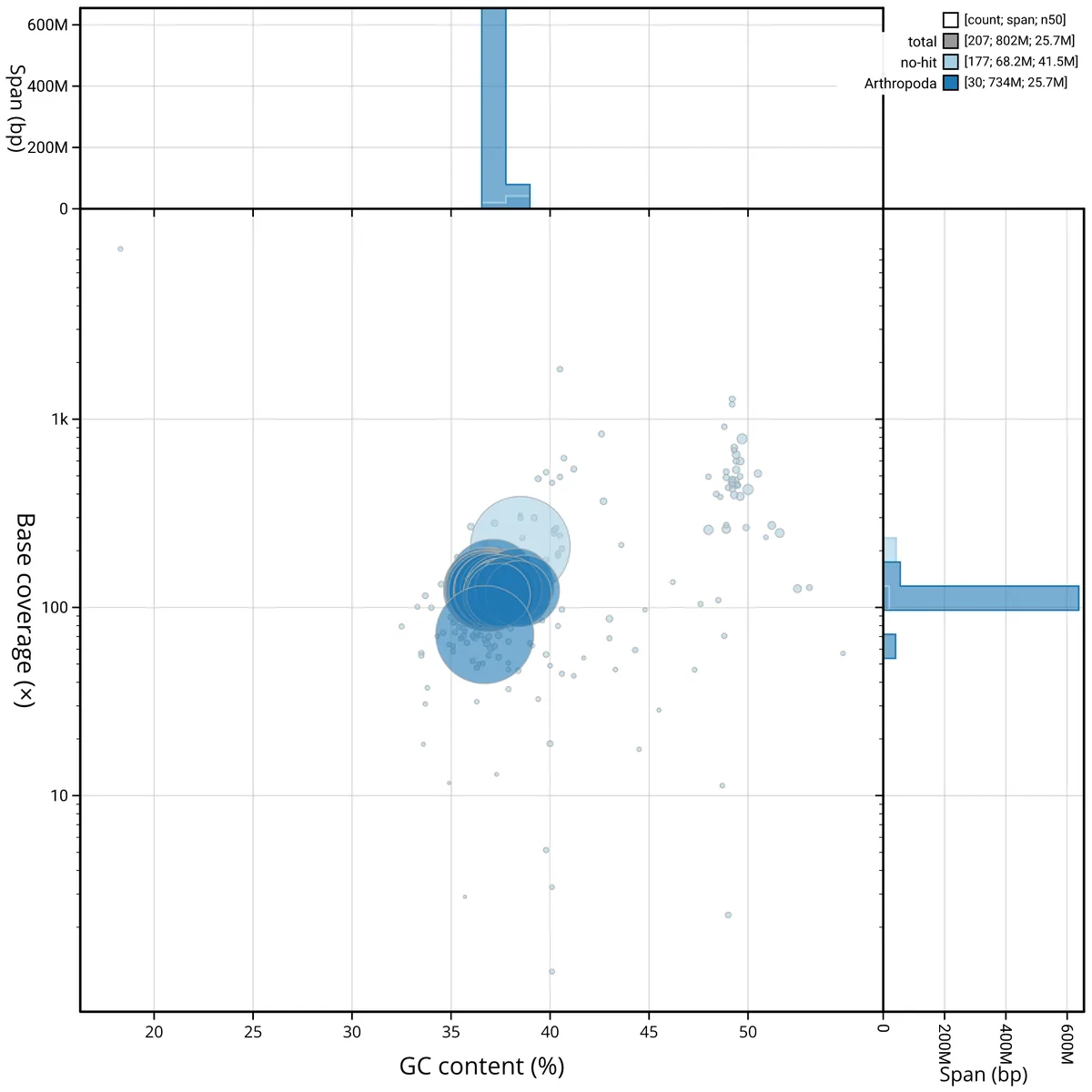
BlobToolKit blob plot for ilHerTars1.hap1.1, the haplotype 1 assembly. The plot shows base coverage (vertical axis) and GC content (horizontal axis). Circles represent scaffolds; marker area is proportional to scaffold length, and colour indicates taxonomic assignment. The histograms along the axes display the total length of sequences distributed across different levels of coverage and GC content. The assembly can be explored interactively in the BlobToolKit viewer.

Table 4 lists the assembly metric benchmarks adapted from Rhie *et al.*. (2021) the Earth BioGenome Project (EBP) Report on Assembly Standards. The three-part EBP metric, calculated for the haplotype 1 assembly, is **6.C.Q67**.

**Table 4.** Earth BioGenome Project summary metrics for the *H. tarsicrinalis* assembly

**Measure**	**Value**	***Benchmark***
EBP summary (haplotype 1)	6.C.Q67	*6.C.Q40*
Contig N50 length	9.59 Mb	*> 1 Mb*
Scaffold N50 length	25.74 Mb	*= chromosome N50*
Consensus quality (QV)	Haplotype 1: 67.7; haplotype 2: 68.4; combined: 68.0	*≥ 40*
*k*-mer completeness	Haplotype 1: 68.43%; Haplotype 2: 64.65%; combined: 99.65%	*> 90%*
BUSCO	Haplotype 1: C:98.8% [S:97.6%, D:1.2%], F:0.1%, M:1.1%, n:5,286; Haplotype 2: C:94.4% [S:93.2%, D:1.3%], F:0.2%, M:5.4%, n:5,286	*S > 90%; D < 5%*
Percentage of assembly assigned to chromosomes	99.02%	*≥ 90%*

**Notes:** The EBP summary uses log10(Contig N50); chromosome-level (C) or log10(Scaffold N50); Q (Merqury QV). BUSCO v5.5.0 using the lepidoptera_odb10 lineage dataset: C=complete; S=single-copy; D=duplicated; F=fragmented; M=missing; n=orthologues.

### Genome annotation report

The *H. tarsicrinalis* genome assembly (GCA_964656175.1) was annotated by Ensembl at the European Bioinformatics Institute (EBI). The annotation comprises 21,818 transcripts from 12,268 protein-coding genes and 1,911 non-coding genes. The average transcript length is 16,281.82 bp, with an average of 1.54 coding transcripts per gene and 6.62 exons per transcript. For further information, please refer to the Ensembl annotation page.

### Data availability

European Nucleotide Archive: Herminia tarsicrinalis. Accession number: PRJEB82330; URL: https://identifiers.org/ena.embl/PRJEB82330. The genome sequence is released openly for reuse. The *H. tarsicrinalis* genome sequencing initiative is part of the Sanger Institute Tree of Life Programme ( PRJEB43745) and Project Psyche ( PRJEB71705). All raw sequence data and the haplotype assemblies have been deposited in INSDC databases. Raw data and assembly accession identifiers are reported in Table 1 and Table 2.

Pipelines used for genome assembly at the WSI Tree of Life are available at https://pipelines.tol.sanger.ac.uk/pipelines. Table 5 lists software versions used in this study.

**Table 5.** Software versions and sources used for *H. tarsicrinalis*

**Software**	**Version**	**Source**
BLAST	2.14.0	https://ftp.ncbi.nlm.nih.gov/blast/executables/blast+/
BlobToolKit	4.3.9	https://github.com/genomehubs/blobtoolkit
BlobTk (hosted BlobDir)	0.5.1	https://github.com/genomehubs/blobtk
BlobTk (snail and blob plot renderer)	0.8.3	https://github.com/genomehubs/blobtk
BUSCO	5.5.0	https://gitlab.com/ezlab/busco
busco-alg-painter	0.1.0	https://github.com/Karenvn/busco-alg-painter
bwa-mem2	2.2.1	https://github.com/bwa-mem2/bwa-mem2
DIAMOND	2.1.8	https://github.com/bbuchfink/diamond
fasta_windows	0.2.4	https://github.com/tolkit/fasta_windows
FastK	1.2	https://github.com/thegenemyers/FASTK
GenomeScope	2.1.0	https://github.com/tbenavi1/genomescope2.0
Gfastats	1.3.6	https://github.com/vgl-hub/gfastats
Hifiasm	0.19.8-r603	https://github.com/chhylp123/hifiasm
HiGlass	1.13.4	https://github.com/higlass/higlass
MerquryFK	1.1.0-c1	https://github.com/thegenemyers/MERQURY.FK
Minimap2	2.24-r1122	https://github.com/lh3/minimap2
MitoHiFi	3.2.2	https://github.com/marcelauliano/MitoHiFi
MultiQC	1.14; 1.17 and 1.18	https://github.com/MultiQC/MultiQC
Nextflow	23.10.0	https://github.com/nextflow-io/nextflow
PretextSnapshot	0.0.6	https://github.com/sanger-tol/PretextSnapshot
PretextView	1.0.3	https://github.com/sanger-tol/PretextView
samtools	1.19.2	https://github.com/samtools/samtools
sanger-tol/ascc	0.1.0	https://github.com/sanger-tol/ascc
sanger-tol/blobtoolkit	0.6.0	https://github.com/sanger-tol/blobtoolkit
sanger-tol/curationpretext	1.4.2	https://github.com/sanger-tol/curationpretext
Seqtk	1.4-r122	https://github.com/lh3/seqtk
Singularity	3.9.0	https://github.com/sylabs/singularity
TreeVal	1.1.1	https://github.com/sanger-tol/treeval
YaHS	1.2a.2	https://github.com/c-zhou/yahs

### Author information

Contributors are listed at the following links:

- Members of *oreina* (Papillons de France)
- Members of the Wellcome Sanger Institute Tree of Life Management, Samples and Laboratory team
- Members of Wellcome Sanger Institute Scientific Operations – Sequencing Operations
- Members of the Wellcome Sanger Institute Tree of Life Core Informatics team
- Members of the Tree of Life Core Informatics collective
- Members of the Project Psyche Community

### Wellcome Sanger Institute – Legal and Governance

The materials that have contributed to this genome note have been supplied by a Tree of Life collaborator. The Wellcome Sanger Institute employs a process whereby due diligence is carried out proportionate to the nature of the materials themselves, and the circumstances under which they have been/are to be collected and provided for use. The purpose of this is to address and mitigate any potential legal and/or ethical implications of receipt and use of the materials as part of the research project, and to ensure that in doing so, we align with best practice wherever possible. The overarching areas of consideration are:

- Ethical review of provenance and sourcing of the material
- Legality of collection, transfer and use (national and international).

Each transfer of samples is undertaken according to a Research Collaboration Agreement or Material Transfer Agreement entered into by the Tree of Life collaborator, Genome Research Limited (operating as the Wellcome Sanger Institute), and in some circumstances, other Tree of Life collaborators.

## References

[ref-1] Allio R. Schomaker-Bastos A. Romiguier J. : MitoFinder: Efficient automated large-scale extraction of mitogenomic data in target enrichment phylogenomics. *Molecular Ecology Resources* ;2020;20(4):892–905. 32243090 10.1111/1755-0998.13160 PMC7497042

[ref-2] Altschul S. F. Gish W. Miller W. : Basic Local Alignment Search Tool. *Journal of Molecular Biology* ;1990;215(3):403–410. 2231712 10.1016/S0022-2836(05)80360-2

[ref-3] Buchfink B. Reuter K. Drost H.-G. : Sensitive protein alignments at tree-of-life scale using DIAMOND. *Nature Methods* ;2021;18(4):366–368. 33828273 10.1038/s41592-021-01101-x PMC8026399

[ref-4] Challis R. Richards E. Rajan J. : BlobToolKit – interactive quality assessment of genome assemblies. *G3: Genes, Genomes, Genetics* ;2020;10(4):1361–1374. 32071071 10.1534/g3.119.400908 PMC7144090

[ref-5] Challis Richard Blaxter Mark : Snail plots are badges of genome assembly quality. *G3 Genes|Genomes|Genetics* ;2026;16(6):jkag074. 41941593 10.1093/g3journal/jkag074 PMC13232491

[ref-6] Cheng H. Jarvis E. D. Fedrigo O. : Haplotype-resolved assembly of diploid genomes without parental data. *Nature Biotechnology* ;2022;40(9):1332–1335. 35332338 10.1038/s41587-022-01261-x PMC9464699

[ref-7] Cheng H. Concepcion G. T. Feng X. : Haplotype-resolved *de novo* assembly using phased assembly graphs with Hifiasm. *Nature Methods* ;2021;18(2):170–175. 33526886 10.1038/s41592-020-01056-5 PMC7961889

[ref-8] Fibiger M. Ronkay L. Yela J. L. : *Noctuidae Europaeae. Rivulinae, Boletobiinae, Hypeninae, Araeopteroninae, Eubleminae, Herminiinae, Phytometrinae, Euteliinae, and Micronoctuidae* . Sorø: Entomological Press;2010.12

[ref-9] Formenti G. Abueg L. Brajuka A. : Gfastats: Conversion, evaluation and manipulation of genome sequences using assembly graphs. *Bioinformatics* ;2022;38(17):4214–4216. 35799367 10.1093/bioinformatics/btac460 PMC9438950

[ref-10] Howard Caroline Denton Amy Jackson Benjamin W. : On the path to reference genomes for all biodiversity: Laboratory protocols and lessons learned from processing over 2,000 species in the Sanger Tree of Life. *GigaScience* ;2025;14 giaf119. Reference Source 10.1093/gigascience/giaf119 PMC12548527 41129326

[ref-11] Howe K. Chow W. Collins J. : Significantly improving the quality of genome assemblies through curation. *GigaScience* ;2021;10(1):giaa153. 33420778 10.1093/gigascience/giaa153 PMC7794651

[ref-12] Kerpedjiev P. Abdennur N. Lekschas F. : HiGlass: Web-based visual exploration and analysis of genome interaction maps. *Genome Biology* ;2018;19(1):125. 30143029 10.1186/s13059-018-1486-1 PMC6109259

[ref-13] Kurtzer G. M. Sochat V. Bauer M. W. : Singularity: Scientific containers for mobility of compute. *PLOS ONE* ;2017;12(5):e0177459. 28494014 10.1371/journal.pone.0177459 PMC5426675

[ref-14] Lees D. C. Natural History Museum Genome Acquisition Lab Darwin Tree of Life Barcoding Collective : The genome sequence of the Plumed Fan-foot, *Polypogon plumigeralis* (Hübner, 1825) (Lepidoptera: Erebidae). *Wellcome Open Research* ;2025;10:619. 41458308 10.12688/wellcomeopenres.25062.1 PMC12743215

[ref-15] Manni Mosè Berkeley Matthew R Seppey Mathieu : BUSCO update: Novel and streamlined workflows along with broader and deeper phylogenetic coverage for scoring of eukaryotic, prokaryotic, and viral genomes. *Molecular Biology and Evolution* ;2021;38(10):4647–4654. 34320186 10.1093/molbev/msab199 PMC8476166

[ref-16] Ranallo-Benavidez Timothy R. Jaron Kai S. Schatz Michael C. : GenomeScope 2.0 and Smudgeplot for reference-free profiling of polyploid genomes. *Nature Communications* ;2020;11(1):1432. 32188846 10.1038/s41467-020-14998-3 PMC7080791

[ref-17] Rao S. S. P. Huntley M. H. Durand N. C. : A 3D map of the human genome at kilobase resolution reveals principles of chromatin looping. *Cell* ;2014;159(7):1665–1680. 25497547 10.1016/j.cell.2014.11.021 PMC5635824

[ref-18] Ratnasingham S. Hebert P. D. N. : BOLD: The Barcode of Life Data System (http://www.barcodinglife.org). *Molecular Ecology Notes* ;2007;7(3):355–364. 18784790 10.1111/j.1471-8286.2007.01678.x PMC1890991

[ref-19] Rhie A. Walenz B. P. Koren S. : Merqury: Reference-free quality, completeness, and phasing assessment for genome assemblies. *Genome Biology* ;2020;21(1):245. 32928274 10.1186/s13059-020-02134-9 PMC7488777

[ref-20] Rhie A. McCarthy S. A. Fedrigo O. : Towards complete and error-free genome assemblies of all vertebrate species. *Nature* ;2021;592(7856):737–746. 33911273 10.1038/s41586-021-03451-0 PMC8081667

[ref-21] Uliano-Silva M. Ferreira J. G. R. N. Krasheninnikova K. : MitoHiFi: A Python pipeline for mitochondrial genome assembly from PacBio high fidelity reads. *BMC Bioinformatics* ;2023;24(1):288. 37464285 10.1186/s12859-023-05385-y PMC10354987

[ref-22] Vasimuddin Md. Misra S. Li H. : Efficient architecture-aware acceleration of BWA-MEM for multicore systems. *2019 IEEE international parallel and distributed processing symposium (IPDPS)* . IEEE.2019.314–324. 10.1109/IPDPS.2019.00041

[ref-23] Wright Charlotte J. Stevens Lewis Mackintosh Alexander : Comparative genomics reveals the dynamics of chromosome evolution in Lepidoptera. *Nature Ecology & Evolution* ;2024;8(4):777–790. 38383850 10.1038/s41559-024-02329-4 PMC11009112

[ref-24] Wright Charlotte J. Wahlberg Niklas Vila Roger : Project Psyche: Reference genomes for all Lepidoptera in Europe. *Trends in Ecology & Evolution* ;2025;40(12):1234–1250. 10.1016/j.tree.2025.10.007 41309387

[ref-25] Zhou C. McCarthy S. A. Durbin R. : YaHS: Yet another Hi-C scaffolding tool. *Bioinformatics* ;2023;39(1):btac808. 36525368 10.1093/bioinformatics/btac808 PMC9848053

