# Supplementary material for: The genome sequence of the Shaded Fan-foot, Herminia tarsicrinalis (Knoch, 1782) (Lepidoptera: Erebidae)
Source: Wellcome Open Res. 2026 Sep 14;11:654. [Version 1] doi: 10.12688/wellcomeopenres.27808.1 (PMC13643135; doi:10.12688/wellcomeopenres.27808.1)
Supplement: Peer review history

# Reviewer report: Reviewer response for version 1

**DOI:** 10.21956/wellcomeopenres.30601.r169431

**Published:** 2026-10-06

**Authors:** Jayan Duminda M Senevirathna

**Affiliations:** 1 Uva Wellassa University, Badulla, Uva Province, Sri Lanka

The genome sequence of the Shaded Fan-foot, *Herminia tarsicrinalis*, has been successfully completed, and the recovery and assembly of its sex chromosomes are particularly noteworthy. Haplotype 1 has been assembled to the chromosome level, which represents an important strength of the study. The BUSCO analysis also indicates high gene completeness (>98%), and approximately 12,268 protein-coding genes have been identified. Overall, these results demonstrate a high-quality and comprehensive genome assembly. Therefore, I consider the manuscript suitable for acceptance in its current form.

Are the datasets clearly presented in a usable and accessible format, and the assembly and annotation available in an appropriate subject-specific repository?

Yes

Are sufficient details of the sequencing and extraction, software used, and materials provided to allow replication by others?

Yes

Are the rationale for sequencing the genome and the species significance clearly described?

Yes

Are the protocols appropriate and is the work technically sound?

Yes

Reviewer Expertise:

Genetics and Biotechnology

I confirm that I have read this submission and believe that I have an appropriate level of expertise to confirm that it is of an acceptable scientific standard.

# Reviewer report: Reviewer response for version 1

**DOI:** 10.21956/wellcomeopenres.30601.r169426

**Published:** 2026-10-05

**Authors:** Rajib Deb

**Affiliations:** 1 ICAR-National Research Centre, Guwahat, Assam, India

In the present context,  a chromosome-level, haplotype-resolved genome assembly of *Herminia tarsicrinalis* (Shaded Fan-foot).  The study combines PacBio HiFi  sequencing with Hi-C chromatin interaction data to generate two haplotype assemblies. The overall sequencing and assembly strategy is technically appropriate and follows current best practices for high-quality eukaryotic genome assembly. The combination of PacBio HiFi reads, Hi-C scaffolding, haplotype-resolved assembly using Hifiasm, YaHS scaffolding, manual curation, Merqury, BUSCO and BlobToolKit provides a strong methodological framework.

 Please clarify the difference between the GenomeScope-estimated genome size (750.1 Mb) and the final haplotype 1 assembly size (801.83 Mb).

 Please provide brief details on how the W and Z chromosomes were identified and distinguished using read depth/copy number and Hi-C evidence.

 Please provide the versions and/or key parameters used for the major assembly tools, particularly Hifiasm, YaHS, MerquryFK and BUSCO.

 The individual haplotypes show relatively low k-mer completeness (68.43% and 64.65%), although the combined assembly reaches 99.65%. Please explain this discrepancy.

Are the datasets clearly presented in a usable and accessible format, and the assembly and annotation available in an appropriate subject-specific repository?

Yes

Are sufficient details of the sequencing and extraction, software used, and materials provided to allow replication by others?

Yes

Are the rationale for sequencing the genome and the species significance clearly described?

Partly

Are the protocols appropriate and is the work technically sound?

Yes

Reviewer Expertise:

Genomics

I confirm that I have read this submission and believe that I have an appropriate level of expertise to confirm that it is of an acceptable scientific standard, however I have significant reservations, as outlined above.

# Reviewer report: Reviewer response for version 1

**Published:** 2026-09-14

**Authors:** Automated Benchmarking

**Affiliations:** 1 Wellcome Sanger Institute, Hinxton, UK

As this article is a Genome Note, Automated Benchmarking has been used to determine whether the reported assembly passes three defined checks established by the Earth BioGenome Project: the raw and assembled data are publicly available, the Earth BioGenome Project three-part metric is met, and the BUSCO score passes the stated threshold. For Herminia tarsicrinalis (ilHerTars1), all three checks passed. Data available: pass. ENA study PRJEB82330, raw-read accessions ERR13946513 and ERR13947542, and assembly accessions GCA_964656175.1 and GCA_964656245.1 are public. Earth BioGenome Project three-part metric: pass. Haplotype 1 is reported as 6.C.Q67, meeting the 6.C.Q40 reference standard. BUSCO: pass. Haplotype 1, which is proposed as the reference assembly for this species, has 98.8% complete BUSCOs (97.6% single-copy and 1.2% duplicated). BUSCO software version: 5.5.0; lineage dataset: lepidoptera_odb10. It meets the target of greater than 90% complete and single-copy BUSCOs. This decision uses values reported in the Genome Note and public accession records.

Reviewer Expertise:

NA

I confirm that I have read this submission and believe that I have an appropriate level of expertise to confirm that it is of an acceptable scientific standard.
